# Autophagy in T Cell Function and Aging

**DOI:** 10.3389/fcell.2019.00213

**Published:** 2019-10-02

**Authors:** Fernando Macian

**Affiliations:** Department of Pathology, Albert Einstein College of Medicine, Bronx, NY, United States

**Keywords:** autophagy, chaperone-mediated autophagy, T cell, aging, immunosenescence

## Abstract

Dysregulation of autophagy with age has been identified as a central mechanism of aging affecting many cells and tissues. T cells do also show decreased activity with age of different autophagic pathways. Here, we will review the current knowledge of the different functions that autophagy has in the regulation of T cell homeostasis, differentiation and function and explore how the age-associated decreased in autophagy activity may contribute to the altered T cell responses that characterize T cell immunosenescence.

## Introduction

The characterization of the molecular mechanisms that regulate autophagy has made possible a better understanding the different roles that this essential degradative process plays in the regulation of the immune response. In the last 15 years, autophagy has emerged as a key regulatory process that not only controls immune cell homeostasis and the response to stress, but also participates in the regulation of a wide array of specific immune functions that include, among others, pathogen elimination, antigen presentation or cell activation and differentiation. Comprehensive reviews covering immune functions of autophagy have been published in the last few years (Deretic et al., 2013; Munz, 2016; Deretic and Levine, 2018; Clarke and Simon, 2019). In this manuscript we will specifically review the current knowledge of the cellular processes that different forms of autophagy modulate in T cells, and will explore how changes in the regulation and activity of those autophagic pathways with age may account for the loss of function that occurs in the T cell compartment and contributes to the overall aging of the immune system.

Three different major forms of autophagy haven been described, namely macroautophagy, microautophgay and chaperone-mediated autophagy (CMA). They differ in their regulation, the nature of the cargo they can degrade and the mechanisms that direct cargo trafficking into the lysosomes. In mammalian cells, microautophagy has not been described in lysosomes but instead it has been reported to occur in late endosomes. Hence it has been termed endosomal microautophagy (Sahu et al., 2011). This form of autophagy involves the incorporation of cytosolic material into the late endosome through an ESCRT complex-dependent formation of invaginations in the endosomal membrane (Sahu et al., 2011). As it has also been described for macroautophagy, non-selective “in-bulk” and selective mechanisms of cargo degradation have been reported, the latter involving the recognition of substrates by the Hsc70 chaperone (Sahu et al., 2011; Morozova et al., 2016). However, whereas a characterization of microautophagy has been elegantly performed in different cell types, including dendritic cells (Sahu et al., 2011), there is yet no information as to the potential role of this form of autophagy in T cells. For macroautophagy and CMA, multiple studies have contributed to our current understanding of the array of functions they regulate in the T cell compartment (Puleston and Simon, 2014; Botbol et al., 2016). In this review, we will describe those processes in which these two forms of autophagy participate to control essential mechanisms of homeostasis, activation and differentiation in T cells; and we will discuss how changes in autophagy activity with age may impact T cell responses, and the implications of those changes for T cell immunosenescence.

## Regulation of Autophagy in T Cells

Several studies have begun to characterize the specific regulation of two different forms of autophagy, macroautophagy and CMA, in peripheral T cells. Macroautophagy involves the sequestration of cytosolic content into *de novo* generated double-membrane vesicles, termed autophagosomes, that eventually fuse with the lysosomes to degrade and recycle their cargo. The regulatory complexes that control the induction and progression of each step in this process have been comprehensively characterized and have been reviewed elsewhere (Mizushima et al., 2011). Regarding the specific signals that are involved in the activation of macroautophagy in T cells, initial studies reported that CD4+ and CD8+ T cells upregulated macroautophagy in response to T cell receptor (TCR) engagement (Li et al., 2006; Pua et al., 2007; Hubbard et al., 2010). As in many other cell types, T cells can induce macroautophagy in response to starvation (Li et al., 2006), however, they are also able to induce autophagy can in response to signaling that regulates T cell activation (Pua et al., 2007; Botbol et al., 2015). Data support, though, that basal and activation-induced macroautophagy likely represent different forms of autophagy, which might respond to different stimuli, target different cargo and have distinct functions. The signaling pathways that underlie the induction of macroautophagy in activated T cells have not been fully characterized yet. It has been proposed that the mitogen-activated protein kinase (MAPK) JNK, which is activated downstream of the TCR, may contribute to the induction of macroautophagy, as chemical inhibition of genetic deletion of JNK1 or JNK2 leads to decreased activation-induced macroautophagy in CD4+ T cells (Li et al., 2006). JNK could induce the expression of autophagy-related (*Atg*) genes through the activation of the transcription factor FoxO1 or, alternatively, it could also cause the dissociation of the Bcl2/Beclin1 complex through direct phosphorylation of Bcl2, leaving Beclin1 free to induce autophagy (Wei et al., 2008; Xu et al., 2011). However, none of these mechanisms have been reported yet to participate in autophagy activation in T cells. A recent study has shown that signaling through cytokine receptors that contain the common gamma chain, including the interleukin (IL)-2 receptor, leads to direct induction of macroautophagy. An effect that is mediated by Janus kinase (JAK) signaling (Botbol et al., 2015). Interestingly, blockade of common gamma chain cytokine signaling in activated CD4+ T cells results in a drastic inhibition of macroautophagy, suggesting that, either in an autocrine or a paracrine manner, engagement of the common gamma chain receptor by cytokines represents a major regulator of macroautophagy induction in activated T cells (Botbol et al., 2015). As opposed to starvation-induced autophagy, activation-induced autophagy appears to be independent on mTOR activity. In these cells, TCR and/or common gamma chain receptor engagement causes concomitant activation of mTOR and macroautophagy. Furthermore, the magnitude of autophagy induction in activated CD4+ T cells is not increased by the mTOR inhibitor rapamycin (Botbol et al., 2015).

Regarding CMA, this form of autophagy is also induced in T cells in response to TCR engagement (Valdor et al., 2014). CMA is a selective form of autophagy that targets cytosolic proteins that present CMA-targeting motifs biochemically related to the KFERQ pentapeptide (Dice, 1990). CMA substrates are recognized by the Hsc70 chaperone and delivered to the lysosomes where they interact with the lysosomal associate membrane protein 2A (LAMP-2A) (Cuervo and Dice, 1996). After substrate binding LAMP-2A forms a multimeric translocation complex that unfolds and delivers the substrate to the lysosomal lumen for degradation assisted by a lysosomal lumen resident form of Hsc70 (Kaushik et al., 2006; Bandyopadhyay et al., 2008). In depth descriptions of the detailed mechanism that govern CMA activity have been recently reviewed (Kaushik and Cuervo, 2018). In T cells, activation of CMA following TCR engagement responds to upregulation of the expression of the LAMP-2A, which is mediated by the increased generation of reactive oxygen species in activated cells (Valdor et al., 2014). A similar regulation of CMA activity has been described in fibroblasts subjected to oxidative stress, which also leads to increased expression of LAMP-2A (Kiffin et al., 2004). In CD4+ T cells, generation of reactive oxygen species (ROS) is coupled to the regulation of intracellular calcium signaling, which has as one of its main targets the transcription factor nuclear factor of activated T cells (NFAT) (Macian, 2005; Kwon et al., 2010; Sena et al., 2013). Interestingly, the promoter of *Lamp2a* becomes a target of NFAT in TCR-stimulated T cells, and the activation-induced expression of that gene is prevented by inhibition of the phosphatase calcineurin, which is responsible for the calcium signaling-mediated dephosphorylation and activation of NFAT (Valdor et al., 2014).

## Functions of Autophagy in T Cells

**FIGURE 1:**
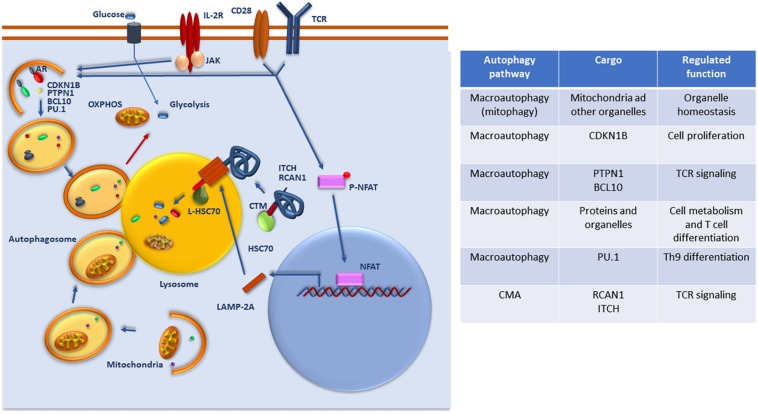
Regulation and function of autophagy in T cells. Whereas basal macroautophagy is a central mechanism of mitochondrial homeostasis, signaling form the TCR, CD28 and/or the IL-2 receptor (IL-2R) activate macroautophagy activity to target specific protein substrates for degradation and regulate glycolytic and oxidative phosphorylation (OXPHOS). Activation of NFAT downstream of the TCR upregulates the expression of LAMP-2A that is targeted to the lysosomes to induce CMA. Selective targeting of specific regulators of TCR signaling that present CMA targeting motifs (CTM) are recognized by Hsc70 and delivered to the lysosome where they will be transported through a translocation complex forms by LAMP-2A multimers into the lysosomal lumen for degradation. A list of the different cargo targeted by macroautophagy and CMA for degradation and the functions that are regulated in T cells through those degradative process is also provided.

Numerous studies carried out over the last 10 years have clearly established that autophagy controls essential programs of homeostasis, survival, activation, differentiation, and metabolic regulation in T cells, constituting a major regulatory mechanism that controls T cell function and fate (Figure 1).

### Autophagy and T Cell Homeostasis

Macroautophagy plays an essential role in the maintenance of T cell homeostasis. Organelle turnover, including mitochondria and endoplasmic reticulum, is severely affected in T cells deficient in key ATG proteins (Pua et al., 2009; Jia and He, 2011; Jia et al., 2011). Mitophagy-regulated mitochondrial turnover is especially important in T cells, as they need to drastically reduce their mitochondrial content when evolving from single positive thymocytes into mature peripheral naïve T cells. Consequently, autophagy-deficient T cells accumulate mitochondria, which are functionally altered. This results in increased ROS accumulation, which translates into higher rates of cell death (Pua et al., 2009). As thymocyte development appears to be essentially unaffected in mice bearing deletions of *Atg* genes in the T cell compartment, increased cell death due to altered mitophagy is likely one of the main factors that account for the markedly reduced numbers of peripheral T cells observed in those mice (Pua et al., 2007; Willinger and Flavell, 2012; Parekh et al., 2013). However, other mechanisms are also likely to contribute to the reduced size of the peripheral T cell population in mice with defective macroautophagy. Increased levels of proapoptotic proteins in T cells may be a consequence not only of increased oxidative stress, but also from a possible role of autophagy in the turnover of some of those proteins, which would also contribute to the increased susceptibility to cell death that occurs the absence of functional macroautophagy (Pua et al., 2007; Kovacs et al., 2012).

### Autophagy and T Cell Activation

Several reports have shown that T cells that lack essential *Atg* genes show reduced proliferative responses to TCR engagement that cannot be overridden by CD28 or IL2-receptor signaling. The mechanisms behind this effect are still not completely understood. Whereas the mitochondrial dysfunction and altered metabolic output observed in T cells from *Atg*-deficient mice are sure to have an negative impact on the responses to antigen, defects in activation-induced proliferation are also observed when autophagy is acutely blocked using inducible deletion of *Atg* genes or utilizing chemical inhibitors (Hubbard et al., 2010). Organelles, and especially mitochondria, appear to be the preferred cargo in autophagosomes found in resting cells, however, activated cells tend to exclude mitochondria from autophagosomes, whose content becomes preferentially cytosolic material (Hubbard et al., 2010). This suggests that selective degradation of cargo may contribute to the regulation of activation-induced responses in T cells. Indeed, several studies have identified selective degradation of inhibitors of cyclin dependent kinases (i.e., CDKN1B) or TCR signaling (i.e., the protein tyrosine phosphatase PTPN1), which also contribute to the reduced proliferative responses observed in autophagy-deficient T cells (Jia et al., 2015; Mocholi et al., 2018). Accumulation of CDKN1B due to reduced macroautophagy-dependent degradation in ATG-7-deficient T cells prevents them from progressing into the cell cycle and results in defective primary responses to pathogen (Jia et al., 2015). Similarly, autophagy-mediated degradation of PTPN1 would eliminate the inhibitory effect of this protein phosphatase on signaling pathways downstream of the TCR and allow for effective T cell activation. Interestingly, the accumulation of PTPN1 on autophagy-deficient CD4+ T cells does not only result in decreased responses to T cell priming but it also renders those cells hyporesponsive to subsequent stimulation, supporting a role for macroautophagy in the regulation of T cell tolerance (Mocholi et al., 2018). In any case, macroautophagy may exert its regulatory control on T cell activation through more complex mechanism that allow for the positive or negative modulation of specific signaling pathways. In this sense, it has been shown that autophagy-mediated degradation of BCL10 is required to modulate TCR-induced NFkB activation in effector but not naïve T cells (Paul et al., 2012). Selective degradation of those proteins should be mediated by autophagy adaptors, such as p62, which are able to recognize modified substrates and bring them to the nascent autophagosome through interaction with autophagosome membrane integral proteins of the LC3/GABARAP family (Paul et al., 2012).

Macroautophagy is not the only form of autophagy that has been shown to modulate T cell activation. As mentioned above, CMA is also induced in activated T cells where it controls TCR signaling through the selective degradation of at least two proteins, RCAN1 and ITCH, that negatively regulate essential signaling pathways activated downstream of the TCR. CMA-mediated degradation of these proteins is required for sustained signaling following TCR engagement. Consequently, mice bearing T cells that are deficient in LAMP-2A, accumulate those proteins and fail to mount effective responses to pathogen infection (Valdor et al., 2014).

### Autophagy and T Cell Metabolism

As a response to starvation, autophagy may provide cells with substrates needed to synthesize new cellular components and obtain energy, thus exerting an important role sustaining the cell’s metabolism. In activated T cells, macroautophagy has also been shown to regulate metabolic pathways to modulate cell function and differentiation (Dowling and Macian, 2018). ATG-7 deficient CD4+ T cells show reduced production of ATP in response to TCR-engagement and both anaerobic glycolysis and mitochondrial respirations appear to be reduced when lysosomal activity is blocked (Hubbard et al., 2010; Mocholi et al., 2018). Recent studies have defined specific links between autophagy and T cell metabolism and identified situations where autophagy-regulation of T cell metabolic activity influences effector functions and cell differentiation. Though it is likely that autophagy may provide substrates to feed distinct metabolic pathways, one of the mechanisms that have been proposed to account for the ability of autophagy to modulate T cell metabolism involves the activation of mTOR and mTOR-regulated metabolic pathways. In activated T cells, the adaptor protein TAX1BP1 promotes induction of macroautophagy to, among other things, generate cysteine and activate mTOR to sustain activation-induced responses (Whang et al., 2017). Interestingly, whereas *Tax1bp1^–/–^* T cells are deficient in autophagy and show reduced glycolytic activity, starvation-induced autophagy does not appear to be compromised, further supporting the concept of the specific characteristics of the increased in macroautophagy activity that follows T cell activation (Hubbard et al., 2010; Whang et al., 2017). However, as we will discuss below, other studies have shown that the ability of macroautophagy to tip the balance between glycolysis and oxidative phosphorylation (OXPHOS) toward the latter in other contexts may underlie its role in promoting the generation of memory T cells during infections with pathogens or the acquisition of defined effector properties in the tumor microenvironment or under inflammatory conditions (Xu et al., 2014; DeVorkin et al., 2019).

Though CMA has been shown to regulate carbohydrate and lipid metabolism in liver and to regulate the levels of several enzymes and key upstream metabolic regulators such as c-Myc, whether this form of autophagy may also modulate T cell metabolism remains to be determined (Schneider et al., 2014; Gomes et al., 2017).

### Autophagy and T Cell Differentiation and Function

Besides its ability to regulate T cell activation, autophagy also participates in determining T cell fate and function. Distinct effects of the loss of *Atg* genes in T cells have been reported in different situations, which might suggest that cooperation between autophagy and context-specific signals received by T cells may determine how autophagy regulates the acquisition of distinct effector functions. In a model of inflammatory bowel disease, mice bearing ATG16L1-deficient T cells develop a more severe disease with increased T helper (Th)2 differentiation and reduced regulatory T cell (Treg) generation, with no major alterations on Th1 and Th17 populations (Kabat et al., 2016). On the other hand, in a mouse model of diet induced steatosis, ATG7-deficient T cells show increased expression of IFN ɣ and IL-17 in both CD4+ and CD8+ T cells, suggesting differentiation bias toward Th1 and Th17 phenotypes in CD4+ T cells (Amersfoort et al., 2018). Furthermore, through regulated degradation of the transcription factor PU.1, autophagy has also been shown to limit Th9 differentiation, and T cells deficient in ATG3 or ATG5 show increased IL-9 production and improved IL-9-mediated tumor control (Rivera Vargas et al., 2017). As mention above, Treg homeostasis appears also to be dependent on the maintenance of functional autophagy. Indeed, specific deletion of *Atg5* or *Atg7* in Tregs results in decreased survival and altered lineage stability of this cell population, that leads to the development of autoimmune inflammatory disease (Wei et al., 2016).

The mechanisms that mediate the roles of autophagy in T cell differentiation are multiple and point to the ability of autophagy to modulate different cellular processes in T cells. Not only direct control of the turnover of specific proteins (e.g., PU.1 in Th9 cells) but also modulation of T cell metabolism have been proposed to underlie many of the effects of autophagy on cell fate determination. For instance, in Tregs, loss of autophagy results in decreased expression of genes involved in fatty acid oxidation and increased expression of glycolytic genes, and triggering of mTORC1 activation. All of this supports glycolytic metabolism, in cells that otherwise should preferentially depend on mitochondrial respiration (Wei et al., 2016). It is important to note that whereas some studies have reported a negative effect of autophagy on mTOR activation, others have seen just the opposite effect (Wei et al., 2016; Whang et al., 2017). Whether these divergent effects are due to different outcomes of autophagy activation on distinct T cell populations (i.e., Tregs vs. effector T cells) or to integration of autophagy-mediated effects with other environmental cues, remains to be determined.

Specific involvement of macroautophagy in CD8+ T cell function has also been reported. Three different studies confirmed that autophagy is required for the effective development of CD8+ T cell memory to pathogen antigens, which was also later confirmed for CD4+ T cells (Chen et al., 2014; Puleston et al., 2014; Xu et al., 2014; Murera et al., 2018). This effect is likely mediated by the ability of autophagy to engage metabolic pathways that support the generation and maintenance of memory T cell populations (Xu et al., 2014). Two recent studies have also addressed how autophagy may participate in regulating the efficacy of anti-tumor CD8+ T cell responses. An increased generation of effector interferon (IFN) ɣ and TNFα-producing CD8+ T cells was observed in tumor-infiltrating lymphocytes in bone marrow chimeras bearing ATG5-deficient donor cells, which led to improved tumor control. This appeared to be a consequence of an increased glycolytic metabolism in autophagy-deficient T cells and to changes in the landscape of histone methylation that affected genes involved in the regulation of T cell activation and metabolism (DeVorkin et al., 2019). A second study showed that elevated potassium levels in the tumor microenvironment resulted in reduced nutrient uptake by CD8+ T cells and the consequent activation of autophagy, which caused metabolic reprograming toward mitochondrial OXPHOS. Increased OXPHOS forced the preferential mitochondrial utilization of the cellular pool of acetyl coenzyme A. Reduced cytosolic and nuclear content of acetyl coenzyme A caused changes in histone acetylation affecting programs of gene expression that eventually led to increased T cell stemness, decreased T cell exhaustion and, consequently, improved tumor control (Vodnala et al., 2019). These data also provide further support to the proposed role of autophagy in the maintenance of stem properties in other cell types (Garcia-Prat et al., 2016). Although both studies point toward the ability of autophagy to reprogram CD8+ T cell metabolism, alter epigenetic marks and limit effector functions, they provide different functional consequences in terms of tumor control resulting from the activation of autophagy in T cells. Though the reason for this discrepancy may reside in the different functional outcomes that may be caused by long-term (knock-out model) or acute loss of autophagy, both studies support, nevertheless, a central role of autophagy in the modulation of anti-tumor CD8+ T cell responses.

## Autophagy and T Cell Aging

**FIGURE 2:**
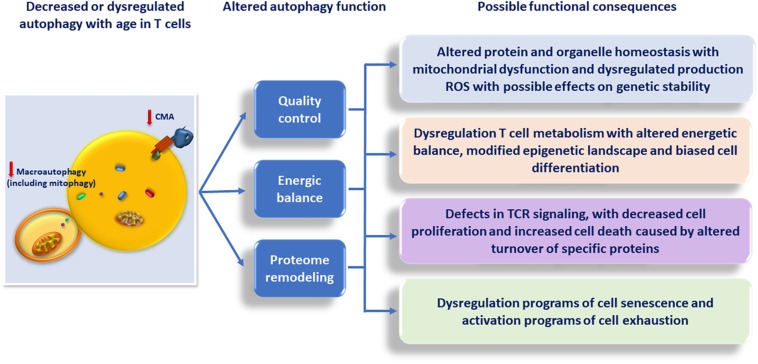
Consequences of the decrease in Macroautophagy and CMA activity with age in T cells.

The wide spectrum of aspects of T cell biology that are regulated by autophagy gives support to the idea that changes of autophagy activity with age may have a significant impact in the quality and magnitude of the T cell response, and contribute to the overall defects in T cell function that characterize immunosenescence. Although we will explore this possibility, it is important, though, to bear in mind that autophagy plays also important roles in many other cells that directly or indirectly regulate T cell responses. These include, among others, participating in the regulation of the response to and the clearance of pathogens in macrophages and other innate immune cells, the regulation of antigen presentation by dendritic cells and epithelial thymic cells or the differentiation of B cells into antibody-producing plasma cells (Gutierrez et al., 2004; Paludan et al., 2005; Pengo et al., 2013; Riffelmacher et al., 2017). A comprehensive evaluation of the effects that altered autophagy with age has, not only in T cells but also in those other immune system cells, should lead to a better understanding of the role of autophagy in T cell immunosenescence (Figure 2).

### Autophagy Activity in Old T Cells

Whereas studies of how microautophagy might be affected during aging are still lacking, it is widely accepted that dysregulation of macroautophagy and CMA occurs in many cell types and tissues with age (Kaushik and Cuervo, 2015). Indeed, changes in the mechanisms that regulate proteostasis, in which autophagy plays an essential role, have been recently defined as one of basic mechanisms responsible for aging (Lopez-Otin et al., 2013; Kennedy et al., 2014). Furthermore, as we have discussed above, autophagy also contributes to the regulation of other basic mechanisms of aging, including modulation of cellular metabolism and nutrient sensing pathways, maintenance of stemness properties in cells, regulation of epigenetic signatures, repair of macromolecular damage and prevention of organelle dysfunction and inflammation or the cellular response to different forms of stress. Both human and mouse models show that macroautophagy and CMA activities decrease with age in T cells. This decrease affects CD4+ and CD8+ T cells and has been reported in naïve and memory cell populations (Phadwal et al., 2012; Cuervo and Macian, 2014; Valdor et al., 2014; Zhang et al., 2016; Raz et al., 2017). The mechanisms responsible for the reduced autophagic activity in T cells are not yet fully understood. Transcriptomic analyses suggest that differences in the expression levels of some *Atg* genes may contribute to limit macroautophagy activity in old T cells (Xiao et al., 2018), although other studies have failed to identify a clear autophagy-related gene signature associated with aging in T cells (Alpert et al., 2019). This may respond to the fact that changes in autophagy with age may not be mostly due to decreased *Atg* gene expression but rather may respond to altered composition of intracellular membranes or defects in signaling. Indeed, altered lysosomal membrane lipid composition in hepatocytes from old mice has been shown to reduce LAMP2-A stability, which leads to decreased CMA activity (Rodriguez-Navarro et al., 2012). Similar changes in lipid composition may also affect autophagosome:lysosome fusion and inhibit macroautophagy (Koga et al., 2010). In T cells, dysregulation of specific pathways may also contribute to the altered activation of autophagy with age. Signaling through the MAPK p38 inhibits autophagy in human CD8+ T cells through direct inhibition of p38IP:ATG9 interaction, which prevents adequate ATG9 intracellular trafficking. This effect is preferentially observed in senescent-like CD45RA+ effector memory CD8+ T cells, which could indicate that defects in autophagy may be more severe in those T cell populations that present a senescent-like exhausted phenotype (Henson et al., 2014). In this sense, autophagy appears to be inducible in aged CD8+ CD28+ T cells from old individuals in response to TCR engagement, but TCR-induced signaling fails to do so in CD8+ CD28− T cells that usually accumulate with age and present a senesce-like phenotype with decreased responsiveness to antigen (Arnold et al., 2014). Given that IL-2 receptor signaling induces macroautophagy in peripheral CD4+ T cells (Botbol et al., 2015), it is likely that the decreased ability to produce this cytokine that characterizes aged T cells may also have a negative effect on their ability to maintain adequate levels of autophagy activity with age. Furthermore, the elevated levels of ROS that may accumulate in old T cells and their inability to modulate production of ROS in response to TCR activation may be behind the reduced induction of *Lamp2a* expression that occurs in aged CD4+ T cells and results in decreased CMA activity (Valdor et al., 2014).

### Functional Consequences of Dysregulated Autophagy in the T Cell Compartment With Age

It is difficult to assess the exact consequences derived from the dysregulation of autophagy activity in T cells with age. Elegant studies have clearly established that, in specific tissues and at the organismal level, restoration of autophagy activity leads to increased function, health-span and live-span in mice (Zhang and Cuervo, 2008; Fernandez et al., 2018). Maybe due to the paucity of available information on the exact molecular mechanism that underlie the changes in autophagy that occur in T cells with age and when they become functionally relevant, those type of studies are still lacking in the T cell compartment. Nevertheless, the characterization of the specific functions of autophagy in T cells and the fact that the consequences of the loss of autophagy in animal models replicate some of the central aspects of the aging phenotype in T cells, provide support to the idea that the loss of autophagy may contribute to the functional consequences of aging in T cells. For instance, accumulation of cellular damage and mitochondrial dysfunction are consequences of autophagy failure that also occur in T cells with age. In other cell types autophagy has been shown to prevent senescence in part due to its ability to maintain mitochondrial homeostasis and prevent oxidative stress (Garcia-Prat et al., 2016). Recently, mitochondrial stress has been proposed to mediate telomere attrition in aged CD8+ T cells, though the authors of this study failed to detect a significant correlation between telomere shortening and decreased autophagy activity (Sanderson and Simon, 2017). As mentioned above, though, studies have shown a clear defect in autophagy in senescent functionally exhausted T cells, which may account for the reduced capacity of those cells to maintain effective responses to antigen. This effect can be mediated, at least in part, by the loss of the ability to adapt the cell’s metabolism to distinct functional and differentiation requirements in the presence of age-associated altered autophagy activity (Henson et al., 2014; Dowling and Macian, 2018). Indeed, a recent study has identified inefficient one carbon metabolism due to mitochondrial dysfunction as a major determinant of defective naïve T cells responses in old mice (Ron-Harel et al., 2018).

Several pieces of evidence support though that decreased autophagy activity has an important impact on the aged T cell function. T cells from old individuals that belong to families with history of extended longevity and healthy aging show preserved autophagy activity in their T cells when compared to age-matched controls (Raz et al., 2017). Improved autophagy correlates with improved function, which may respond, among other possible mechanisms, to higher levels of expression of autophagy-associated genes (Raz et al., 2017; Xiao et al., 2018). A reduced ability of T cells to generate effective memory in response to pathogens or vaccination is one of the defining characteristics of immunosenescence, which is mainly due to intrinsic T cell defects. As discussed earlier, mice bearing autophagy deficient CD8+ T cells fail to properly develop and maintain populations of memory T cells that can protect against pathogen re-challenge (Puleston et al., 2014; Xu et al., 2014). Interestingly, administration of spermidine to those mice, a compound that has the ability to activate autophagy, restores the ability of old mice to mount protective memory responses to influenza challenge (Puleston et al., 2014).

Supporting that the age-induced decline in the activity of other forms of autophagy has also a direct effect on T cell function, it has been shown that when CMA activity is boosted by promoting increased expression of *Lamp2a ex vivo*, responses to TCR stimulation of old CD4+ T cells are restored to levels similar to those observed in young T cells (Valdor et al., 2014).

### Can Autophagy Be a Therapeutic Target to Boost T Cell Responses?

Loss of proteostasis represents a basic mechanism of aging that affects many organs and systems. Autophagy activity declines with age in many cell types including T cells, and though the magnitude of the impact on T cell responses of the age associated decline in autophagy remains to be fully evaluated, the possibility of restoring autophagy activity to improve not only proteostasis but also organelle homeostasis and regulate cell metabolism, offers a very attractive approach to rejuvenate cells and improve their function. Restoration of macroautophagy or CMA in mouse models of aging has been shown to improve cell and organ function and increase health and life span (Zhang and Cuervo, 2008; Fernandez et al., 2018). Furthermore, many of the interventions that have been shown to increase health-span in animal models, such as caloric restriction or rapamycin, are also well characterized inducers of autophagy (Cox and Mattison, 2009; Harrison et al., 2009; Escobar et al., 2019). Can we attain a similar beneficial effect in T cells? Available data might support so. As discussed above, activation of autophagy by administration of spermidine to old mice restores their ability to generate effective CD8+ T cell memory (Puleston et al., 2014). Furthermore, it was first shown in mice that inhibition of mTORC1 results in enhanced quality and magnitude of the CD8+ T cell memory response to viral infection (Araki et al., 2009). Later studies performed in old humans confirmed that inhibition of mTOR resulted in clearly enhanced responses to influenza vaccine and also in reduced expression of exhaustion markers such a PD-1 (Mannick et al., 2014). A later study by the same group has shown that low doses of mTOR inhibitors result not only in better responses to influenza vaccination but in an overall decrease in the incidence of infectious disease in the elderly (Mannick et al., 2018). Whether these effects are mediated through activation of autophagy remains to be determined. It is important also to consider that though those drugs may activate autophagy in T cells and directly enhance their function, it is also likely they may induce autophagy in other cell types, such as dendritic cells, macrophages of B cells, which should also contribute to an overall improve immune response in individuals treated with those drugs.

Other more general interventions such as increased exercise, caloric restriction or intermittent fasting, do also have the potential to activate autophagy and have also been shown to modulate the immune response (Martinez-Lopez et al., 2017; Escobar et al., 2019).

## Conclusion

Autophagy plays an central role in the regulation of T cell homeostasis and function. Dysregulation of autophagy with age constitutes a basic mechanism of aging and it contributes to the age-associated loos of function in many cells and tissues, including T cells. We are beginning to appreciate the functional consequences and the mechanisms that account for the loss of autophagy regulation in different T cell populations with age. The possibility of improving autophagy activity to boost T cell function offers a very attractive therapeutic approach to enhance immune response in the elderly. The development of more specific drugs that might be able to restore the activity of defined autophagic pathway may, therefore, offer new possibilities to potentiate the response to vaccines, decrease the incidence of infectious disease and possibly enhance the anti-tumor T cell response.

## Author Contributions

The author confirms being the sole contributor of this work and has approved it for publication.

## Conflict of Interest

The author declares that the research was conducted in the absence of any commercial or financial relationships that could be construed as a potential conflict of interest.
